# Association of Alcohol Consumption After Development of Heart Failure With Survival Among Older Adults in the Cardiovascular Health Study

**DOI:** 10.1001/jamanetworkopen.2018.6383

**Published:** 2018-12-28

**Authors:** Justin S. Sadhu, Eric Novak, Kenneth J. Mukamal, Jorge R. Kizer, Bruce M. Psaty, Phyllis K. Stein, David L. Brown

**Affiliations:** 1 Cardiovascular Division, Washington University School of Medicine, St Louis, Missouri; 2 Division of General Medicine and Primary Care, Beth Israel Deaconess Medical Center, Boston, Massachusetts; 3 Department of Medicine, Harvard Medical School, Boston, Massachusetts; 4 Cardiology Section, San Francisco Veterans Affairs Health Care System, San Francisco, California; 5 Department of Medicine, University of California, San Francisco; 6 Department of Epidemiology and Biostatistics, University of California, San Francisco; 7 Cardiovascular Health Research Unit, Departments of Medicine, Epidemiology and Health Services, University of Washington, Seattle; 8 Group Health Research Institute, Group Health Cooperative, Seattle, Washington

## Abstract

**Importance:**

More than 1 million older adults develop heart failure annually. The association of alcohol consumption with survival among these individuals after diagnosis is unknown.

**Objective:**

To determine whether alcohol use is associated with increased survival among older adults with incident heart failure.

**Design, Setting, and Participants:**

This prospective cohort study included 5888 community-dwelling adults aged 65 years or older who were recruited to participate in the Cardiovascular Health Study between June 12, 1989, and June 1993, from 4 US sites. Of the total participants, 393 individuals had a new diagnosis of heart failure within the first 9 years of follow-up through June 2013. The study analysis was performed between January 19, 2016, and September 22, 2016.

**Exposures:**

Alcohol consumption was divided into 4 categories: abstainers (never drinkers), former drinkers, 7 or fewer alcoholic drinks per week, and more than 7 drinks per week.

**Primary Outcomes and Measures:**

Participant survival after the diagnosis of incident heart failure.

**Results:**

Among the 393 adults diagnosed with incident heart failure, 213 (54.2%) were female, 339 (86.3%) were white, and the mean (SD) age was 78.7 (6.0) years. Alcohol consumption after diagnosis was reported in 129 (32.8%) of the participants. Across alcohol consumption categories of long-term abstainers, former drinkers, consumers of 1-7 drinks weekly and consumers of more than 7 drinks weekly, the percentage of men (32.1%, 49.0%, 58.0%, and 82.4%, respectively; *P* < .001 for trend), white individuals (78.0%, 92.7%, 92.0%, and 94.1%, respectively, *P* <. 001 for trend), and high-income participants (22.0%, 43.8%, 47.3%, and 64.7%, respectively; *P* < .001 for trend) increased with increasing alcohol consumption. Across the 4 categories, participants who consumed more alcohol had more years of education (mean, 12 years [interquartile range (IQR), 8.0-10.0 years], 12 years [IQR, 11.0-14.0 years], 13 years [IQR, 12.0-15.0 years], and 13 years [IQR, 12.0-14.0 years]; *P* < .001 for trend). Diabetes was less common across the alcohol consumption categories (32.1%, 26.0%, 22.3%, and 5.9%, respectively; *P* = .01 for trend). Across alcohol consumption categories, there were fewer never smokers (58.3%, 44.8%, 35.7%, and 29.4%, respectively; *P* < .001 for trend) and more former smokers (34.5%, 38.5%, 50.0%, and 52.9%, respectively; *P* = .006 for trend). After controlling for other factors, consumption of 7 or fewer alcoholic drinks per week was associated with additional mean survival of 383 days (95% CI, 17-748 days; *P* = .04) compared with abstinence from alcohol. Although the robustness was limited by the small number of individuals who consumed more than 7 drinks per week, a significant inverted U-shaped association between alcohol consumption and survival was observed. Multivariable model estimates of mean time from heart failure diagnosis to death were 2640 days (95% CI, 1967-3313 days) for never drinkers, 3046 days (95% CI, 2372-3719 days) for consumers of 0 to 7 drinks per week, and 2806 (95% CI, 1879-3734 days) for consumers of more than 7 drinks per week (*P* = .02). Consumption of 10 drinks per week was associated with the longest survival, a mean of 3381 days (95% CI, 2806-3956 days) after heart failure diagnosis.

**Conclusions and Relevance:**

These findings suggest that limited alcohol consumption among older adults with incident heart failure is associated with survival benefit compared with long-term abstinence. These findings suggest that older adults who develop heart failure may not need to abstain from moderate levels of alcohol consumption.

## Key Points

**Question:**

What is the association of alcohol consumption after the diagnosis of heart failure with survival among older adults?

**Findings:**

In this cohort study of 393 older adults with incident heart failure, consumption of 7 or fewer alcoholic drinks per week was significantly associated with increased survival compared with abstinence from alcohol, after controlling for other factors.

**Meaning:**

Limited alcohol consumption among older adults with incident heart failure may be associated with increased survival.

## Teaser

This cohort study investigates whether the consumption of alcohol after the diagnosis of heart failure among older adults who participated in the Cardiovascular Health Study was associated with increased survival compared with abstinence.

## Introduction

The association between alcohol consumption and heart failure (HF) is complex. Although excessive alcohol consumption may result in HF due to an alcoholic cardiomyopathy,^1,2^ moderate alcohol consumption has been associated with a reduction in the risk of incident HF.^3,4,5,6^ However, a more recent analysis suggests that alcohol consumption of more than approximately 7 drinks per week is associated with an increased hazard of HF, with a neutral association observed with less alcohol consumption.^7^ In contrast to the conflicting data regarding the association between previous alcohol consumption and development of HF, to our knowledge, there are no data regarding the safety of alcohol consumption in patients following a new diagnosis of HF. Previous studies examining alcohol use in adults with HF have yielded mixed results, but they were limited to individuals with prevalent disease.^8,9,10,11^ Thus, the possibility of a survivor bias influencing these results cannot be excluded. With more than 1 million new cases of HF diagnosed annually in the United States among individuals 55 years or older,^12^ data regarding the association of alcohol consumption in this population is lacking. We sought to examine the association between alcohol consumption and survival following the diagnosis of HF in a cohort of older adults in a community setting.

## Methods

This investigation conforms to the principles outlined in the Declaration of Helsinki.^13^ Because Cardiovascular Health Study data were deidentified, the Washington University Office of Human Research Protection, St Louis, Missouri, granted an exemption from institutional review board review. The study followed the Strengthening the Reporting of Observational Studies in Epidemiology (STROBE) reporting guideline.^14^ The study analysis was performed between January 19, 2016, and September 22, 2016.

The Cardiovascular Health Study is a prospective cohort study of 5888 black and white adults aged 65 years or older in the United States. Detailed descriptions of the Cardiovascular Health Study have been previously published.^15,16^ Participants were recruited from 4 US sites: Forsyth County, North Carolina; Sacramento County, California; Pittsburgh, Pennsylvania; and Washington County, Maryland. They received baseline clinical examinations from 1989 through 1993, followed by 9 yearly clinic visits.

Baseline alcohol consumption was determined at the initial visit, and former drinkers were differentiated from abstainers (never drinkers).^5^ Alcohol consumption was ascertained in a similar manner during follow-up years 2 through 5 and years 7 through 9 but was not obtained in follow-up years 1 and 6 for administrative reasons.^5^ To allow for assessment of alcohol consumption during the first visit after the diagnosis of HF, only patients with incident HF diagnosed during the first 10 years of the Cardiovascular Health Study were included. An alcoholic beverage was defined as a 12-oz beer, a 6-oz glass of wine, or a shot (1.5 ounces) of liquor, and alcohol consumption was reported by each participant as the number of alcoholic beverages consumed per week.

Clinical events were ascertained by telephone calls twice per year, with follow-up available through June 30, 2013. Diagnoses of HF were adjudicated by the Cardiovascular Health Study Events Committee and required (1) a diagnosis of HF by a physician and medical treatment for congestive HF or (2) imaging evidence of cardiomegaly and pulmonary edema by chest radiography or dilated ventricle and reduced systolic function by echocardiography or contrast ventriculography.

Based on the first available measure of alcohol consumption following a new diagnosis of HF, study participants were divided into 4 categories: (1) abstainers (never drinkers), (2) former drinkers, (3) persons consuming 7 or fewer alcoholic drinks per week, and (4) persons consuming more than 7 drinks per week. A cutoff of 7 drinks per week was chosen because this is the recommended maximum alcohol consumption for this age population.^17,18^

### Statistical Analysis

Cross-sectional associations were assessed using analysis of variance, Fisher exact test, or Kruskal-Wallis tests, as appropriate. Trends among abstainers, participants consuming 7 or fewer drinks per week, and those consuming more than 7 drinks per week were evaluated by Spearman correlation coefficient for continuous or ordinal variables, the Mantel-Haenszel χ^2^ test for linear association for ordinal variables, and the Cochran-Armitage trend test for dichotomous variables. We excluded former alcohol drinkers from the analyses of trends because they do not constitute an ordinal category of alcohol consumption. Former drinkers may have quit drinking in response to changes in health (sick quitters), and we were interested in examining how continued alcohol consumption after diagnosis of HF was related to survival compared with long-term abstinence.

We used multiple linear regression to model the association between alcohol consumption as a categorical variable and subsequent survival (time from diagnosis of HF to death or last follow-up)^19^ after adjustment for age, sex, race/ethnicity, educational level, income, smoking status, marital status, hypertension, diabetes, stroke, myocardial infarction, atrial fibrillation, angiotensin-converting enzyme inhibitor use, β-blocker use, systolic blood pressure, diastolic blood pressure, body mass index, 15-ft walk time, glomerular filtration rate, and the presence of left ventricular hypertrophy reported on the electrocardiogram. β-Blocker and angiotensin-converting enzyme inhibitor use was ascertained in the first visit following the diagnosis of HF. Hypertension and weight were determined from the visit immediately before HF diagnosis. Glomerular filtration rate, smoking status, marital status, systolic blood pressure, diastolic blood pressure, 15-ft walk time, left ventricular hypertrophy reported by electrocardiogram, and height were ascertained from the closest visit before or after the diagnosis of HF. Alcohol use assessment was ascertained at the first visit after the diagnosis except among 22 participants who had missing data. In those cases, we used the value immediately before the diagnosis was made, to which the mean difference in alcohol consumption before and after the diagnosis derived from individuals with both values was added.

A 2-sided *P* < .05 defined statistical significance. Multiple linear regression has previously been used in this data set to model associations between exposures and survival.^19^ Model assumptions were evaluated by examining residual plots. After excluding former drinkers, we also assessed alcohol use as a continuous variable, including a quadratic term to test for an inverted U-shaped association between alcohol use and survival and adjustment for the same covariates. Mean estimates of time from incident HF to death along with 95% CIs were subsequently derived from the multivariable models. Sequential methods were used to impute missing baseline data.^20^ All analyses were conducted using SAS, version 9.4 (SAS Institute Inc).

## Results

Of 393 individuals with incident HF, 213 (54.2%) were female, 339 (86.3%) were white, and the mean (SD) age was 78.7 (6.0) years. In addition, 168 participants (42.7%) were abstainers from alcohol use, 96 (24.4%) were former drinkers, 112 (28.5%) were current drinkers consuming 7 or fewer drinks per week, and 17 (4.3%) were current drinkers consuming more than 7 drinks per week (Table). All but 22 of 393 individuals (5.6%) died during follow-up.

**Table.** Patient Characteristics at Diagnosis of Heart Failure

Variable	Overall (N = 393)	Long-term Abstinence (n = 168)	Former Drinker (n = 96)	≤7 Current Drinks/wk (n = 112)	>7 Current Drinks/wk (n = 17)	*P* Value (Differences Among All Groups)	*P* Value for Trend^a^
Age, mean (SD), y	78.7 (6.0)	78.4 (6.2)	78.8 (5.8)	79.2 (5.9)	77.1 (5.1)	.46	.32
Male, No. (%)	180 (45.8)	54 (32.1)	47 (49.0)	65 (58.0)	14 (82.4)	<.001	<.001
Race/ethnicity, No. (%)							
White	339 (86.3)	131 (78.0)	89 (92.7)	103 (92.0)	16 (94.1)	.002	<.001^b^
Black	51 (13.0)	36 (21.4)	6 (6.2)	8 (7.1)	1 (5.9)		
Other	3 (0.8)	1 (0.6)	1 (1.0)	1 (0.9)	0		
Marital status, No. (%)							
Married	207 (52.7)	80 (47.6)	54 (56.2)	65 (58.0)	8 (47.1)	.48	.18^c^
Widowed	162 (41.2)	76 (45.2)	35 (36.5)	42 (37.5)	9 (52.9)		
Other	24 (6.1)	12 (7.1)	7 (7.3)	5 (4.5)	0		
Educational level, median (IQR), y	12 (10.0-14.0)	12 (8.0-12.0)	12 (11.0-14.0)	13 (12.0-15.0)	13 (12.0-14.0)	<.001	<.001
Income, No. (%), $							
<16 000	156 (39.7)	90 (53.6)	34 (35.4)	30 (26.8)	2 (11.8)	<.001	<.001
16 000-24 999	94 (23.9)	41 (24.4)	20 (20.8)	29 (25.9)	4 (23.5)		
≥25 000	143 (36.4)	37 (22.0)	42 (43.8)	53 (47.3)	11 (64.7)		
Hypertension, No. (%)	274 (69.7)	122 (72.6)	64 (66.7)	76 (67.9)	12 (70.6)	.73	.45
Diabetes mellitus, No. (%)	105 (26.7)	54 (32.1)	25 (26.0)	25 (22.3)	1 (5.9)	.06	.01
History of myocardial infarction, No. (%)	85 (21.6)	29 (17.3)	31 (32.3)	22 (19.6)	3 (17.6)	.04	.68
Atrial fibrillation, No. (%)	78 (19.8)	30 (17.9)	15 (15.6)	29 (25.9)	4 (23.5)	.23	.13
History of stroke, No. (%)	42 (10.7)	18 (10.7)	7 (7.3)	13 (11.6)	4 (23.5)	.23	.35
BMI, mean (SD)	27.6 (5.2)	28.2 (6.0)	27.7 (5.4)	26.8 (3.4)	26.0 (4.4)	.09	.14
Smoking status, No. (%)							
Never	186 (47.3)	98 (58.3)	43 (44.8)	40 (35.7)	5 (29.4)	.002	<.001
Former	160 (40.7)	58 (34.5)	37 (38.5)	56 (50.0)	9 (52.9)		.006
Current	47 (12.0)	12 (7.1)	16 (16.7)	16 (14.3)	3 (17.6)		.03
Systolic blood pressure, mean (SD), mm Hg	137.6 (24.2)	137.2 (24.7)	138.6 (25.6)	138.4 (22.7)	131.4 (21.3)	.69	.86
Diastolic blood pressure, mean (SD), mm Hg	67.4 (13.2)	67.1 (12.4)	69.0 (15.5)	66.3 (12.6)	68.0 (11.6)	.51	.75
Angiotensin-converting enzyme inhibitor, No. (%)	167 (42.5)	65 (38.7)	46 (47.9)	50 (44.6)	6 (35.3)	.44	.51
β-Blocker, No. (%)	77 (19.6)	31 (18.5)	24 (25.0)	20 (17.9)	2 (11.8)	.47	.67
15-ft Walk time, median (IQR), s	6 (5.0-8.0)	6 (5.0-9.0)	6 (5.0-7.0)	5 (4.0-7.0)	6 (4.0-9.0)	.001	<.001
GFR, mean (SD), ml/min	65 (20)	66 (20)	63 (21)	64 (18)	66 (20)	.76	.48
Left ventricular hypertrophy by ECG, No. (%)	63 (16.0)	32 (19.0)	11 (11.5)	19 (17.0)	1 (5.9)	.3	.31

Abbreviations: BMI, body mass index (calculated as weight in kilograms divided by height in meters squared); ECG, electrocardiogram; GFR, glomerular filtration rate; IQR, interquartile range.

^a^ Trend among long-term abstinence, 7 or fewer current drinks per week, and more than 7 current drinks per week.

^b^ White vs black or other.

^c^ Married vs widowed or other.

Across alcohol consumption categories of long-term abstainers, former drinkers, consumers of 1-7 drinks weekly and consumers of more than 7 drinks weekly, the percentage of men (54 of 168 [32.1%], 47 of 96 [49.0%], 65 of 112 [58.0%], and 14 of 17 [82.4%], respectively; *P* < .001 for trend), white individuals (131 [78.0%], 89 [92.7%], 103 [92.0%], and 16 [94.1%], respectively, *P* <. 001 for trend), and high-income participants (37 [22.0%], 42 [43.8%], 53 [47.3%], and 11 [64.7%], respectively; *P* < .001 for trend) increased with increasing alcohol consumption. Across the 4 categories, participants who consumed more alcohol had more years of education (mean, 12 years [interquartile range (IQR), 8.0-10.0 years], 12 years [IQR, 11.0-14.0 years], 13 years [IQR, 12.0-15.0 years], and 13 years [IQR, 12.0-14.0 years]; *P* < .001 for trend). Diabetes was less common across the alcohol consumption categories (54 [32.1%], 25 [26.0%], 25 [22.3%], and 1 [5.9%], respectively; *P* = .01 for trend). Across alcohol consumption categories, there were fewer never smokers (98 [58.3%], 43 [44.8%], 40 [35.7%], and 5 [29.4%], respectively; *P* < .001 for trend) and more former smokers (58 [34.5%], 37 [38.5%], 56 [50.0%], and 9 [52.9%], respectively; *P* = .006 for trend) (Table). The mean 15-ft walk time was lowest (5 seconds [IQR, 4.0-7.0 seconds]), indicating better fitness, in the moderate consumption group (≤7 drinks/wk). The median survival after diagnosis of HF was 7.5 years (IQR, 4.3-10.9 years). After controlling for other factors, consumption of 7 or fewer drinks per week was associated with significantly longer survival following the diagnosis of HF compared with abstinence (survival of an additional 383 days, 95% CI, 17-748 days; *P* = .04). Although robustness was limited by the small number of individuals who consumed more than 7 drinks per week, a significant inverted U-shaped association between alcohol consumption and survival was observed (Figure). Multivariable model estimates of mean time from heart failure diagnosis and death were 2640 days (95% CI, 1967-3313 days) for never drinkers, 3046 days (95% CI, 2372-3719 days) for consumers of 1 to 7 drinks per week, and 2806 days (95% CI, 1879-3734 days) for consumers of more than 7 drinks per week (*P* = .02) (Figure). Consumption of 10 drinks per week was associated with the longest survival, a mean of 3381 days (95% CI, 2806-3956 days) after heart failure diagnosis.

**Figure.**
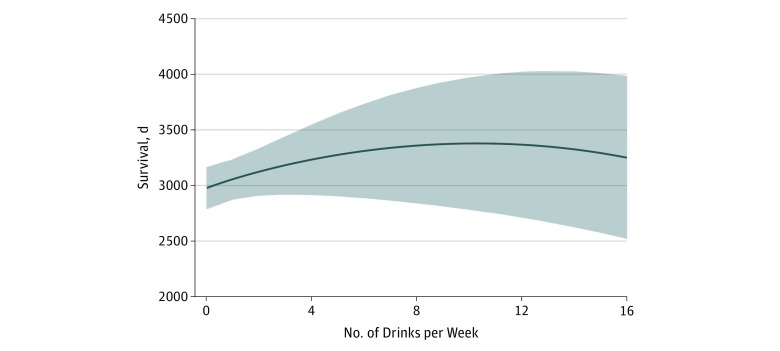
Projected Survival After Diagnosis of Heart Failure by Weekly Alcohol Consumption Mean estimates of time from incident heart failure to death or last follow-up were derived from a multivariable model that included linear and quadratic terms for alcohol consumption and that was adjusted for age, sex, race/ethnicity, educational level, income, smoking status, marital status, hypertension, diabetes, stroke, myocardial infarction, atrial fibrillation, angiotensin-converting enzyme inhibitor and β-blocker use, systolic blood pressure, diastolic blood pressure, body mass index, 15-ft walk time, glomerular filtration rate, and presence of left ventricular hypertrophy on electrocardiography.

## Discussion

Among community-dwelling older adults, alcohol consumption of 7 or fewer drinks per week after the diagnosis of HF was associated with longer survival compared with abstinence, after adjustment for important covariates, including age, sex, race/ethnicity, educational level, income, smoking status, marital status, hypertension, diabetes, stroke, myocardial infarction, atrial fibrillation, angiotensin-converting enzyme inhibitor use, β-blocker use, systolic blood pressure, diastolic blood pressure, body mass index, 15-ft walk time, glomerular filtration rate, and presence of left ventricular hypertrophy reported on electrocardiography. Although mixed associations of alcohol use and mortality have been observed in individuals with prevalent left ventricular dysfunction or HF,^8,9,10,11^ the current study is unique to date because it analyzed survival in an inception cohort with HF and it distinguished between former drinkers and abstainers.

Our findings of increased survival among community-dwelling older adults with HF and moderate alcohol consumption are consistent with observations reported in other studies in the population of patients without HF.^21,22,23^ Moderate alcohol consumption appears to be protective against coronary atherosclerotic events, perhaps because of increases in high-density lipoprotein concentration, reductions in plasminogen activator, increases in plasminogen activator inhibitor activities, and improvement in insulin sensitivity.^24,25^ Moreover, polyphenols found in beer and wine increase nitric oxide concentration, which may have benefits in adults with HF.^25^

Current guidelines provide little guidance with respect to recommendations regarding alcohol consumption in patients with HF: the 2010 Heart Failure Society of America guideline^26^ recommends limiting alcohol consumption to no more than 2 drinks per day in men and 1 drink per day in women, whereas the American College of Cardiology Foundation/American Heart Association and the European Society of Cardiology guidelines^27,28^ are largely silent about the topic.

Our findings have potential implications for the 1 million adults aged more than 55 years who are newly diagnosed with HF each year in the United States.^12^ In older patients with incident HF who consumed alcohol before their diagnosis, limited alcohol consumption after the diagnosis appears to be safe. Previous studies of patients with prevalent HF have found lower mortality^8,9^ and improved perceived and objective health status^10^ with light to moderate alcohol consumption. By focusing on individuals with newly diagnosed HF rather than prevalent HF, we reduced the likelihood that any observed associations between alcohol consumption and survival were influenced by survivor bias. Nevertheless, our findings should not be used to recommend that individuals who are newly diagnosed with HF should initiate alcohol consumption if they had formerly been abstainers. Likewise, patients with suspected alcohol-induced cardiomyopathy should continue to abstain from alcohol consumption.

## Limitations

Our study had several limitations. We used a single measure of alcohol consumption after the diagnosis of HF and could not adjust for the association between subsequent changes in alcohol consumption levels and survival. We did not have information about the cause of HF or about left ventricular ejection fraction and cannot comment whether the associations of alcohol consumption and survival differed among patients with reduced vs preserved left ventricular ejection fraction. Given the limitations of our sample size, we did not perform subgroup analyses by age or sex. In addition, the group of patients who consumed more than 7 drinks per week after the diagnosis of HF consisted of only 17 individuals, limiting any conclusions that can be drawn about higher levels of alcohol consumption. Despite attempts to control for important covariates, we cannot exclude the possibility of residual confounding between alcohol use and other favorable prognostic factors influencing its observed association with improved survival.

## Conclusions

Alcohol use is common among older adults who have been diagnosed with HF. These findings suggest that limited alcohol consumption among older adults with incident HF is associated with survival benefit compared with long-term abstinence. Given the possibility of health benefits with limited alcohol consumption following this diagnosis, further efforts to determine optimal levels of alcohol consumption in adults with HF and whether this differs by age, sex, left ventricular ejection fraction, or cause of HF are warranted.

## References

[1] Kupari M, Koskinen P, Suokas A Left ventricular size, mass and function in relation to the duration and quantity of heavy drinking in alcoholics. Am J Cardiol. 1991;67(4):-. doi:10.1016/0002-9149(91)90559-4 1825010

[2] Urbano-Marquez A, Estruch R, Navarro-Lopez F, Grau JM, Mont L, Rubin E The effects of alcoholism on skeletal and cardiac muscle. N Engl J Med. 1989;320(7):409-415. doi:10.1056/NEJM198902163200701 2913506

[3] Walsh CR, Larson MG, Evans JC, Alcohol consumption and risk for congestive heart failure in the Framingham Heart Study. Ann Intern Med. 2002;136(3):181-191. doi:10.7326/0003-4819-136-3-200202050-00005 11827493

[4] Abramson JL, Williams SA, Krumholz HM, Vaccarino V Moderate alcohol consumption and risk of heart failure among older persons. JAMA. 2001;285(15):1971-1977. doi:10.1001/jama.285.15.1971 11308433

[5] Bryson CL, Mukamal KJ, Mittleman MA, The association of alcohol consumption and incident heart failure: the Cardiovascular Health Study. J Am Coll Cardiol. 2006;48(2):305-311. doi:10.1016/j.jacc.2006.02.066 16843180

[6] Gonçalves A, Claggett B, Jhund PS, Alcohol consumption and risk of heart failure: the Atherosclerosis Risk in Communities Study. Eur Heart J. 2015;36(15):939-945. doi:10.1093/eurheartj/ehu514 25602025 PMC4481602

[7] Wood AM, Kaptoge S, Butterworth AS, ; Emerging Risk Factors Collaboration/EPIC-CVD/UK Biobank Alcohol Study Group Risk thresholds for alcohol consumption: combined analysis of individual-participant data for 599 912 current drinkers in 83 prospective studies. Lancet. 2018;391(10129):1513-1523. doi:10.1016/S0140-6736(18)30134-X 29676281 PMC5899998

[8] Cooper HA, Exner DV, Domanski MJ Light-to-moderate alcohol consumption and prognosis in patients with left ventricular systolic dysfunction. J Am Coll Cardiol. 2000;35(7):1753-1759. doi:10.1016/S0735-1097(00)00625-2 10841221

[9] Petrone AB, Gaziano JM, Djoussé L Alcohol consumption and risk of death in male physicians with heart failure. Am J Cardiol. 2014;114(7):1065-1068. doi:10.1016/j.amjcard.2014.07.021 25129877 PMC4163088

[10] Cosmi F, Di Giulio P, Masson S, ; GISSI-HF Investigators Regular wine consumption in chronic heart failure: impact on outcomes, quality of life, and circulating biomarkers. Circ Heart Fail. 2015;8(3):428-437. doi:10.1161/CIRCHEARTFAILURE.114.002091 25925415

[11] GargiuloG, TestaG, CacciatoreF, Moderate alcohol consumption predicts long-term mortality in elderly subjects with chronic heart failure. J Nutr Health Aging. 2013;17(5):480-485. doi:10.1007/s12603-012-0430-4 23636551

[12] Benjamin EJ, Virani SS, Callaway CW, ; American Heart Association Council on Epidemiology and Prevention Statistics Committee and Stroke Statistics Subcommittee Heart disease and stroke statistics—2018 update: a report from the American Heart Association. Circulation. 2018;137(12):e67-e492. doi:10.1161/CIR.0000000000000558 29386200

[13] World Medical Association World Medical Association Declaration of Helsinki: ethical principles for medical research involving human subjects. JAMA. 2013;310(20):2191-2194. doi:10.1001/jama.2013.281053 24141714

[14] von Elm E, Altman DG, Egger M, Pocock SJ, Gøtzsche PC, Vandenbroucke JP; STROBE Initiative The Strengthening the Reporting of Observational Studies in Epidemiology (STROBE) statement: guidelines for reporting observational studies. PLoS Med. 2007;4(10):e296. doi:10.1371/journal.pmed.0040296 17941714 PMC2020495

[15] Fried LP, Borhani NO, Enright P, The Cardiovascular Health Study: design and rationale. Ann Epidemiol. 1991;1(3):263-276. doi:10.1016/1047-2797(91)90005-W 1669507

[16] Ives DG, Fitzpatrick AL, Bild DE, Surveillance and ascertainment of cardiovascular events: the Cardiovascular Health Study. Ann Epidemiol. 1995;5(4):278-285. doi:10.1016/1047-2797(94)00093-9 8520709

[17] National Institute on Alcohol Abuse and Alcoholism Older adults. https://www.niaaa.nih.gov/alcohol-health/special-populations-co-occurring-disorders/older-adults. Accessed July 28, 2018.

[18] Oslin D, Maust D Addictions In: Medina-Walpole A, Pacala JT, Potter JF, eds. Geriatrics Review Syllabus: A Core Curriculum in Geriatric Medicine. 9th ed New York, NY: American Geriatrics Society; 2016.

[19] Alshawabkeh LI, Yee LM, Gardin JM, Years of able life in older persons–the role of cardiovascular imaging and biomarkers: the Cardiovascular Health Study. J Am Heart Assoc. 2015;4(4):e001745. doi:10.1161/JAHA.114.001745 25907126 PMC4579951

[20] Arnold AM, Kronmal RA Multiple imputation of baseline data in the cardiovascular health study. Am J Epidemiol. 2003;157(1):74-84. doi:10.1093/aje/kwf156 12505893

[21] Xi B, Veeranki SP, Zhao M, Ma C, Yan Y, Mi J Relationship of alcohol consumption to all-cause, cardiovascular, and cancer-related mortality in US adults. J Am Coll Cardiol. 2017;70(8):913-922. doi:10.1016/j.jacc.2017.06.054 28818200

[22] Di Castelnuovo A, Costanzo S, Bagnardi V, Donati MB, Iacoviello L, de Gaetano G Alcohol dosing and total mortality in men and women: an updated meta-analysis of 34 prospective studies. Arch Intern Med. 2006;166(22):2437-2445. doi:10.1001/archinte.166.22.2437 17159008

[23] Ronksley PE, Brien SE, Turner BJ, Mukamal KJ, Ghali WA Association of alcohol consumption with selected cardiovascular disease outcomes: a systematic review and meta-analysis. BMJ. 2011;342:d671. doi:10.1136/bmj.d671 21343207 PMC3043109

[24] Brien SE, Ronksley PE, Turner BJ, Mukamal KJ, Ghali WA Effect of alcohol consumption on biological markers associated with risk of coronary heart disease: systematic review and meta-analysis of interventional studies. BMJ. 2011;342:d636. doi:10.1136/bmj.d636 21343206 PMC3043110

[25] de Gaetano G, Costanzo S, Di Castelnuovo A, Effects of moderate beer consumption on health and disease: a consensus document. Nutr Metab Cardiovasc Dis. 2016;26(6):443-467. doi:10.1016/j.numecd.2016.03.007 27118108

[26] Lindenfeld J, Albert NM, Boehmer JP, ; Heart Failure Society of America HFSA 2010 comprehensive heart failure practice guideline. J Card Fail. 2010;16(6):e1-e194. doi:10.1016/j.cardfail.2010.04.004 20610207

[27] Yancy CW, Jessup M, Bozkurt B, ; American College of Cardiology Foundation; American Heart Association Task Force on Practice Guidelines 2013 ACCF/AHA guideline for the management of heart failure: a report of the American College of Cardiology Foundation/American Heart Association Task Force on Practice Guidelines. J Am Coll Cardiol. 2013;62(16):e147-e239. doi:10.1016/j.jacc.2013.05.019 23747642

[28] Ponikowski P, Voors AA, Anker SD, ; ESC Scientific Document Group 2016 ESC guidelines for the diagnosis and treatment of acute and chronic heart failure: the Task Force for the diagnosis and treatment of acute and chronic heart failure of the European Society of Cardiology (ESC) developed with the special contribution of the Heart Failure Association (HFA) of the ESC. Eur Heart J. 2016;37(27):2129-2200. doi:10.1093/eurheartj/ehw128 27206819

