# Data Error in Abstract

**DOI:** 10.1001/jamanetworkopen.2018.8276

**Published:** 2019-02-01

In the Original Investigation titled “Association of Alcohol Consumption After Development of Heart Failure With Survival Among Older Adults in the Cardiovascular Health Study,”^1^ published December 28, 2018, in the Results section of the Abstract, the alcohol consumption category in the multivariable model estimate of mean time from heart failure diagnosis to death for consumers of “0 to 7 drinks per week” should have been reported as “1 to 7 drinks per week.” This article was corrected online.^1^
